# Coronary CT Angiography-derived Fractional Flow Reserve Testing in Patients with Stable Coronary Artery Disease: Recommendations on Interpretation and Reporting

**DOI:** 10.1148/ryct.2019190050

**Published:** 2019-11-21

**Authors:** Bjarne L. Nørgaard, Timothy A. Fairbairn, Robert D. Safian, Mark G. Rabbat, Brian Ko, Jesper M. Jensen, Koen Nieman, Kavitha M. Chinnaiyan, Niels Peter Sand, Hitoshi Matsuo, Jonathon Leipsic, Gilbert Raff

**Affiliations:** From the Department of Cardiology, Aarhus University Hospital, Palle Juul-Jensens Boulevard 99, 8200 Aarhus, Denmark (B.L.N., J.M.J.); Liverpool Centre for Cardiovascular Science, Liverpool Heart and Chest Hospital, Liverpool, England (T.A.F.); Department of Cardiology and Radiology, Beaumont Health System, Royal Oak, Mich (R.D.S., K.M.C., G.R.); Division of Cardiology, Loyola University Chicago, Chicago, Ill (M.G.R.); Monash Cardiovascular Research Centre, Monash University, Monash University and MonashHeart, Monash Health, Clayton, Victoria, Australia (B.K.); Department of Cardiology, Stanford University School of Medicine, Palo Alto, Calif (K.N.); Department of Cardiology, Hospital of Southwest DK, Esbjerg, Denmark (N.P.S.); Department of Cardiovascular Medicine, Gifu Heart Center, Gifu, Japan (H.M.); and Department of Medical Imaging, St Paul’s Hospital, Vancouver, Canada (J.L.).

## Abstract

Noninvasive fractional flow reserve derived from coronary CT angiography (FFR_CT_) is increasingly used in patients with coronary artery disease as a gatekeeper to the catheterization laboratory. While there is emerging evidence of the clinical benefit of FFR_CT_ in patients with moderate coronary disease as determined with coronary CT angiography, there has been less focus on interpretation, reporting, and integration of FFR_CT_ results into routine clinical practice. Because FFR_CT_ analysis provides a plethora of information regarding pressure and flow across the entire coronary tree, standardized criteria on interpretation and reporting of the FFR_CT_ analysis result are of crucial importance both in context of the clinical adoption and in future research. This report represents expert opinion and recommendation on a standardized FFR_CT_ interpretation and reporting approach.

Published under a CC BY 4.0 license.

**Summary**

Expert opinion and recommendation was given by an independent group of physicians on a standardized interpretation and reporting approach for CT-derived fractional flow reserve testing supported by years of clinical experience.

**Key Points**

■ Standardized criteria on interpretation and reporting of CT-derived fractional flow reserve (FFR_CT_) analysis results are of importance both in context of their clinical adoption and in future research.
■ Use of the FFR_CT_ value 10–20 mm distal to the lower border of the stenosis for decision making is recommended.
■ We recommend for clinical decision making a dichotomous interpretation strategy to be considered only in lesions with FFR_CT_ greater than 0.80 or lower than or equal to 0.75, whereas, in patients with FFR_CT_ ranging between 0.76 and 0.80, additional risk stratification information is needed.
■ The results of FFR_CT_ must be evaluated in their clinical context, taking into account patient symptoms, the coronary anatomy, and suitability of revascularization.

## Introduction

Since the first study on coronary CT angiography–derived fractional flow reserve (CT FFR) diagnostic performance by Koo and colleagues in 2011 (1), an abundance of data pertaining to this modality has been published. Several tools have been introduced for the calculation of CT FFR (1–3); however, the majority of existing evidence and clinical experience is based on the HeartFlow FFR_CT_ method (HeartFlow, Redwood City, Calif), which is the only CT FFR cleared by the United States Food and Drug Administration (4) and endorsed by the National Institute for Health and Care Excellence in the United Kingdom (5). Comprehensive reviews of the principle of FFR_CT_ have been described previously (6–8). FFR_CT_ assessment is increasingly used in mainstream clinical practice (9–14) and is likely to further expand with the increased utilization of coronary CT angiography as a first-line test in patients suspected of having coronary artery disease (CAD). While there has been much focus on the diagnostic performance and potential clinical utility of FFR_CT_ in patients with moderate CAD (9–18), there has been less focus on interpretation, reporting, and integration of FFR_CT_ results into routine clinical practice (19). A broadly adopted standardized FFR_CT_ interpretation and reporting approach providing rich and consistent information may facilitate more appropriate clinical implementation and stimulate further high-quality research. Thus, this report, which was written by an independent group of physicians with years of clinical experience with FFR_CT_, proposes standardized criteria for FFR_CT_ interpretation and reporting for application in clinical practice and for clinical research.

## FFR versus FFR_CT_

FFR_CT_ provides simultaneous calculation of pressure and flow across the entire coronary tree (Fig 1). In contrast, information pertaining to invasively measured FFR is only available in vessels that have been interrogated with the pressure wire, which is typically decided during invasive coronary angiography at the discretion of the interventionists (20). While anatomic percentage of stenosis is evaluated at the location of the lesion, invasive FFR is typically measured by positioning the pressure sensor in the distal part of the vessel and then manually pulling the pressure sensor back to the ostium to assess the distribution of abnormal epicardial resistance along the course of the vessel (20). In both invasive FFR and FFR_CT_, the distal values in any given vessel reflect the cumulative pressure loss and impact of all disease proximal to the measurement location. Values obtained by both techniques may vary depending on the measurement location within a vessel. Accordingly, in vessels that have been assessed using both techniques, if the measurement locations of invasive FFR and FFR_CT_ are not matched, their values can be different and may not closely correlate.

**Figure 1a:**
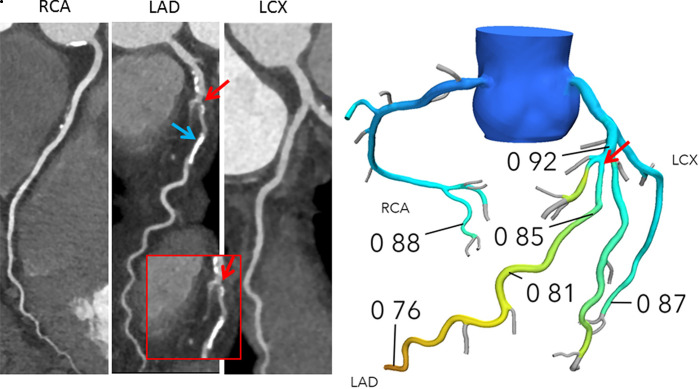
**(a)** Interpretation of FFR_CT_ results in a 65-year-old woman with typical angina. Agatston score, 333. Left: Coronary CT angiography curved multiplanar reconstructions demonstrate a 50%–69% proximal left anterior descending artery (LAD) stenosis (red arrow) and in the mid-LAD, nonobstructive diffuse disease. The blue arrow indicates where the lesion-specific FFR_CT_ value was assessed. Right: In the FFR_CT_ three-dimensional model, the FFR_CT_ value 16 mm distal to the stenosis was 0.85, indicating that the lesion did not cause significant pressure loss. However, FFR_CT_ was significantly low (0.76) in the terminal vessel segments. **(b)** Interpretation of FFR_CT_ results in a 63-year-old man with atypical angina. Agatston score, 245. Left: Coronary CT angiographic images demonstrate a 50%–69% proximal LAD stenosis (red arrow). The blue arrow indicates where the lesion-specific FFR_CT_ value was assessed. Right: In the FFR_CT_ three-dimensional model, the FFR_CT_ value 14 mm distal to the stenosis indicated that the lesion was hemodynamically significant with a value of 0.69. Coronary CT angiography and FFR_CT_ reporting are demonstrated in Figure 4. LCX = left circumflex coronary artery, RCA = right coronary artery.

**Figure 1b:**
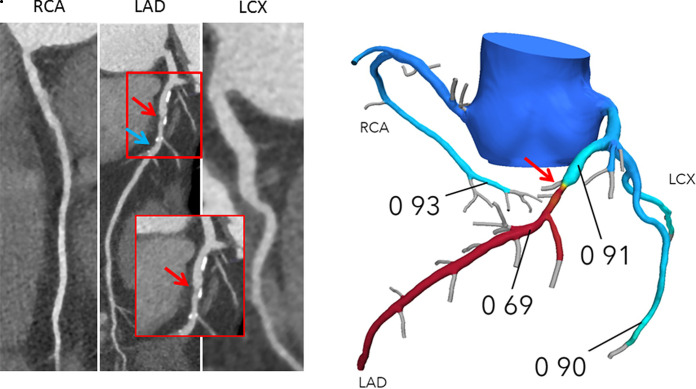
**(a)** Interpretation of FFR_CT_ results in a 65-year-old woman with typical angina. Agatston score, 333. Left: Coronary CT angiography curved multiplanar reconstructions demonstrate a 50%–69% proximal left anterior descending artery (LAD) stenosis (red arrow) and in the mid-LAD, nonobstructive diffuse disease. The blue arrow indicates where the lesion-specific FFR_CT_ value was assessed. Right: In the FFR_CT_ three-dimensional model, the FFR_CT_ value 16 mm distal to the stenosis was 0.85, indicating that the lesion did not cause significant pressure loss. However, FFR_CT_ was significantly low (0.76) in the terminal vessel segments. **(b)** Interpretation of FFR_CT_ results in a 63-year-old man with atypical angina. Agatston score, 245. Left: Coronary CT angiographic images demonstrate a 50%–69% proximal LAD stenosis (red arrow). The blue arrow indicates where the lesion-specific FFR_CT_ value was assessed. Right: In the FFR_CT_ three-dimensional model, the FFR_CT_ value 14 mm distal to the stenosis indicated that the lesion was hemodynamically significant with a value of 0.69. Coronary CT angiography and FFR_CT_ reporting are demonstrated in Figure 4. LCX = left circumflex coronary artery, RCA = right coronary artery.

## FFR_CT_ Interpretation

As for CT angiography, FFR_CT_ interpretation should be performed by the local imaging experts determined by level of clinical knowledge and practical experience with the technique. This may include cardiologists and/or radiologists. It is recommended that downstream management decision making beyond FFR_CT_ takes into account both the clinical scenario (symptoms, risk profile, and/or comorbid conditions) and the coronary anatomy.

### Evaluation of CT Angiography and Lesion Location

The first step in the interpretation of FFR_CT_ is to re-examine the original coronary CT angiography study with particular focus on the location and severity of detailed anatomic lesions (Table). Because FFR_CT_ declines along the length of the vessel with serial focal lesions or areas of diffuse disease, it is important to correlate the pressure loss to specific lesions, which can only be established by direct comparison between the CT angiography lesion location and the FFR_CT_ three-dimensional coronary tree model in relation to identifiable vessel landmarks, such as origin, branches, and segments. It is recommended that this first step be performed by using the Society of Cardiovascular Computed Tomography (SCCT) coronary segmentation model (21).

**Table:**
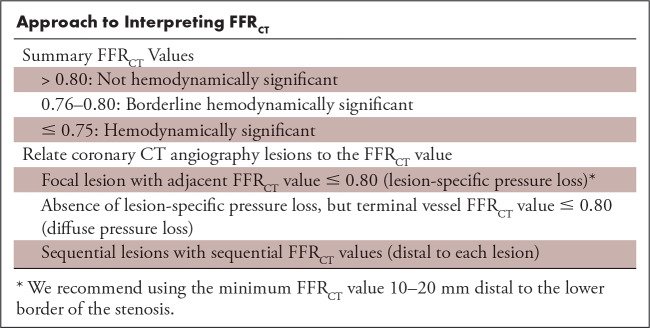
Approach to Interpreting FFR_CT_

### FFR_CT_ Threshold

**Figure 2:**
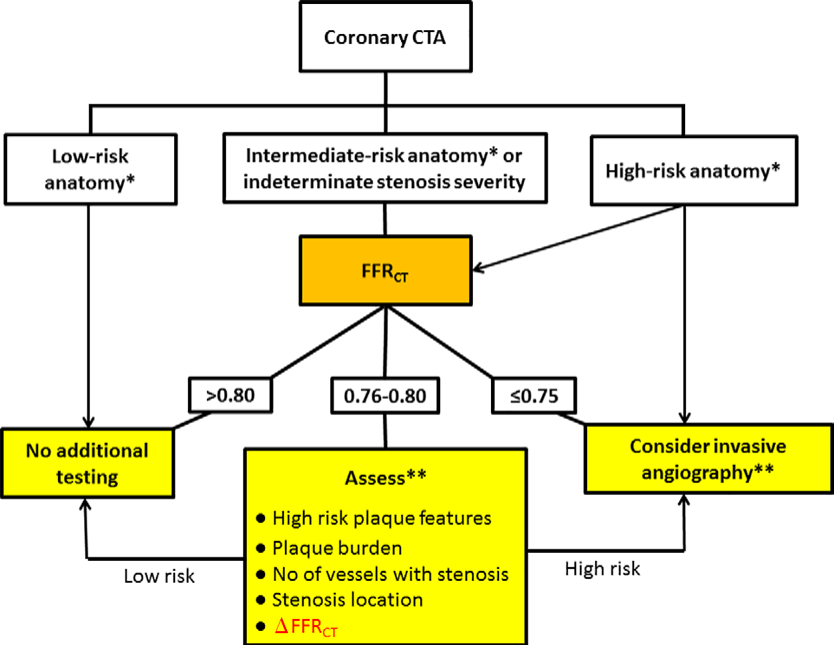
FFR_CT_ appropriateness and interpretation recommendation. * = Low risk: patients either without coronary disease or with maximum stenosis less than 30%. Intermediate risk: patients with one or more intermediate range stenosis (30%–69%). High risk: patients with left main, three-vessel disease or stenosis 70% or greater. Anatomic characteristics beyond stenosis severity, patient symptoms, and suitability of revascularization may influence decisions on management after coronary CT angiography (CTA). ** = Posttest risk stratification: Test results must always be evaluated in their clinical context, taking into account patient symptoms and preferences as well as high-risk anatomic features and likelihood of revascularization.

There is high per-patient and per-vessel agreement between FFR_CT_ and invasive FFR using the threshold of 0.80 for both techniques (1,15–18). An FFR_CT_ value greater than 0.80 indicates that the lesion is unlikely to be hemodynamically significant and that the patient can be safely treated with optimal medical treatment without further downstream testing (1,12–18,22,23). A poststenotic FFR_CT_ value less than or equal to 0.80 indicates the possibility of hemodynamic significance (1,15–18). The use of this dichotomous FFR_CT_ threshold to guide treatment decisions, namely to avoid further downstream testing or consider invasive angiography and revascularization, remains controversial, as it is well known from the invasive literature that the greatest benefit of revascularization is obtained in patients with the most severe pressure loss (24,25). We recommend a dichotomous interpretation strategy to be considered in lesions with FFR_CT_ greater than 0.80 or less than or equal to 0.75 (ie, values >0.80 are “normal” and values ≤0.75 are associated with high likelihood of hemodynamic significance) (Table, Figure 2). Several factors support this strategy. First, FFR_CT_ values are lower than measured FFR (with a bias ranging between 0.03 and 0.05) (16,18). Second, among patients with FFR_CT_ values less than or equal to 0.80, there is a graded correlation between FFR_CT_ and invasive FFR, with the highest FFR_CT_ uncertainty in the range between 0.76 and 0.80 and the highest agreement when FFR_CT_ is less than or equal to 0.75 (12,18). Third, FFR_CT_, similar to FFR, exhibits a continuous relationship between its numerical value and clinical outcomes, with the worst outcome at lower FFR_CT_ values (14,22,23). Finally, symptomatic patients with moderate CAD determined at CT angiography and FFR_CT_ values greater than 0.80 and in whom invasive angiography is deferred have a favorable prognosis (12,14,22,23).

Clinical decision making in patients with FFR_CT_ ranging between 0.76 and 0.80 is nuanced and may benefit from consideration of additional risk stratification information (Fig 2). Identifying patients at incrementally higher cardiovascular risk, who may benefit from an early coronary angiography approach, can be done by assessing several factors: high-risk plaque features (low attenuation, positive remodeling, napkin-ring sign) (26–28), plaque burden (27,28), stenosis location (proximal vs distal; main vessel vs side branch) (25,29,30), vessel territory (left anterior descending artery [LAD] vs non-LAD) (29), ratio of coronary vessel volume to myocardial mass (31), and/or the translesional FFR_CT_ gradient (ΔFFR_CT_) (32). It is the opinion of the present author group that in certain instances with FFR_CT_ values less than or equal to 0.75 (eg, small vessels, distal lesions, side branches), patients may be treated with optimal medical therapy without referral to invasive angiography as a first-line strategy (14,33).

In a recent retrospective study, a large pressure drop (ΔFFR_CT_ ≥ 0.06) was a stronger predictor of culprit lesions for future acute coronary syndromes than FFR_CT_ measured distal to the lesion alone (32). Ongoing studies are assessing the potential diagnostic value of ΔFFR_CT_ in clinical practice. Overall, the results of FFR_CT_, as for invasive FFR, must always be evaluated in their clinical context, taking into account patient symptoms and comorbid conditions, which inform the goals of coronary intervention, in combination with the coronary anatomy and suitability of revascularization.

### Standardized Interpretation of Hemodynamically Significant Lesions

**Figure 3:**
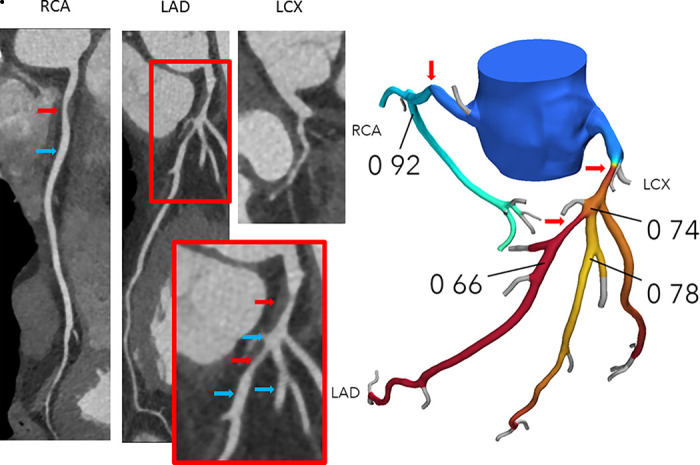
FFR_CT_ assessment in vessels with serial lesions in a 53-year-old man with typical angina. Agatston score, 0. Left: Coronary CT angiography curved multiplanar reconstructions demonstrate a proximal 60% right coronary artery (RCA) stenosis (red arrow) and two serial stenoses in the left anterior descending artery (LAD) (one lesion in the proximal segment with 70% or greater diameter stenosis, and a 50%–69% diameter stenosis lesion distal to the takeoff of the second diagonal [red arrows]). Blue arrows indicate where the FFR_CT_ values were assessed. Right: In the FFR_CT_ three-dimensional model, the FFR_CT_ value 10 mm distal to the proximal LAD stenosis was 0.74 and thus had hemodynamic significance, whereas FFR_CT_ 15 mm distal to the second LAD stenosis was 0.66. FFR_CT_ 10 mm distal to the lower border of the proximal RCA stenosis was 0.92, thus this lesion had low likelihood of being hemodynamically significant. Of note, pressure recovery was observed in the proximal part of the second diagonal with a step-up in FFR_CT_ from 0.74 in the LAD to 0.78 when moving downstream the diagonal branch. LCX = left circumflex coronary artery.

In patients with CAD, as for measured FFR, FFR_CT_ values decline from the ostium to the distal vessel irrespective of the vessel territory, stenosis severity, and location (14,19,34–36). In FFR practice, it is advised that the FFR value within the throat of the lesion (which may correspond to the minimum FFR_CT_ value) is not used clinically and that the pressure is assessed at least 2–3 cm distal to the stenosis of interest (20). Likewise, for clinical decision making, we recommend using the FFR_CT_ value distal to the lesion. With the interactive three-dimensional coronary model tool (HeartFlow), it is possible to obtain multiple values across the vessel. Hence, after localizing the stenosis, the vessel should be serially interrogated downstream from the lesion. Notably, the FFR_CT_ value may transiently rise immediately after the stenosis because of poststenotic vessel dilatation, resulting in reduced flow velocity and pressure recovery (Fig 3). In a recent study, it was suggested that a reliable location at which to assess FFR_CT_ was 1 cm distal to the end of a stenosis (36). For clinical decision making, we recommend using the FFR_CT_ value 1–2 cm distal to the lower border of the stenosis, avoiding the pressure recovery phenomenon.

### Distal Vessel FFR_CT_ Values

FFR_CT_ provides simultaneous computation of pressure and flow in the entire coronary tree, thus exposing both lesion-specific pressure as well as nadir FFR_CT_ values across the coronary system, which in various settings may drop less than or equal to 0.80 (14,19,34–36) (Fig 1). Low terminal vessel FFR_CT_ values (rather than a value distal to stenosis) may include effects unrelated to the stenosis (19,35–37). These low values remote from a focal lesion may be due to diffuse CAD or reflect the sum of serial flow-limiting lesions (35–37). In recent studies, 35%–44% of patients with stable CAD and terminal vessel FFR_CT_ values less than or equal to 0.80 were reclassified as negative when the FFR_CT_ point of reading was 1–2 cm distal to stenosis (14,35). In one observational single-center study, the intermediate follow-up clinical outcome was favorable in patients with terminal FFR_CT_ values less than or equal to 0.80 who were treated with optimal medical treatment (14). In vessels without a significant pressure loss within 2 cm distal to the lesion of interest, but with FFR_CT_ values less than or equal to 0.80 in nearby (eg, mid coronary) segments, we recommend assessment for extent of upstream disease including both CT angiography and FFR_CT_. FFR_CT_ values less than or equal to 0.80 in such circumstances may be clinically relevant (especially when present distal to a lesion in a proximal segment supplying a large myocardial territory). The group recognizes that more research is needed, particularly in large vessels that have discordance between lesion-specific FFR_CT_ and values taken 2 cm beyond an upstream lesion.

### Serial Lesions

The individual contribution of a given lesion in the event of serial stenosis cannot be assessed with FFR_CT_, similar to measured FFR, in any straightforward way because of the complex physiologic interplay between stenoses (Fig 3). At present, there is no accepted way to identify the lesion that contributes most to this cumulative pressure loss. Intuitively, the intrinsic impact of a given lesion should relate to ΔFFR_CT_ of that individual lesion, and previous data have in fact demonstrated excellent correlation between ΔFFR_CT_ and invasive ΔFFR (38). However, in a recent study, it was demonstrated that ΔFFR_CT_ (as well as ΔFFR) may underestimate the physiologic contribution of stenosis in vessels with serial lesions (39). An interactive revascularization FFR_CT_-based planner tool (HeartFlow) may more accurately predict the invasive FFR contribution of each stenosis in serial CAD (39). The ongoing Precise Percutaneous Coronary Intervention plan (P^3^) study (ClinicalTrials.gov: NCT03782688) investigates the diagnostic value of the FFR_CT_ revascularization planner tool.

## FFR_CT_ Reporting

**Figure 4:**
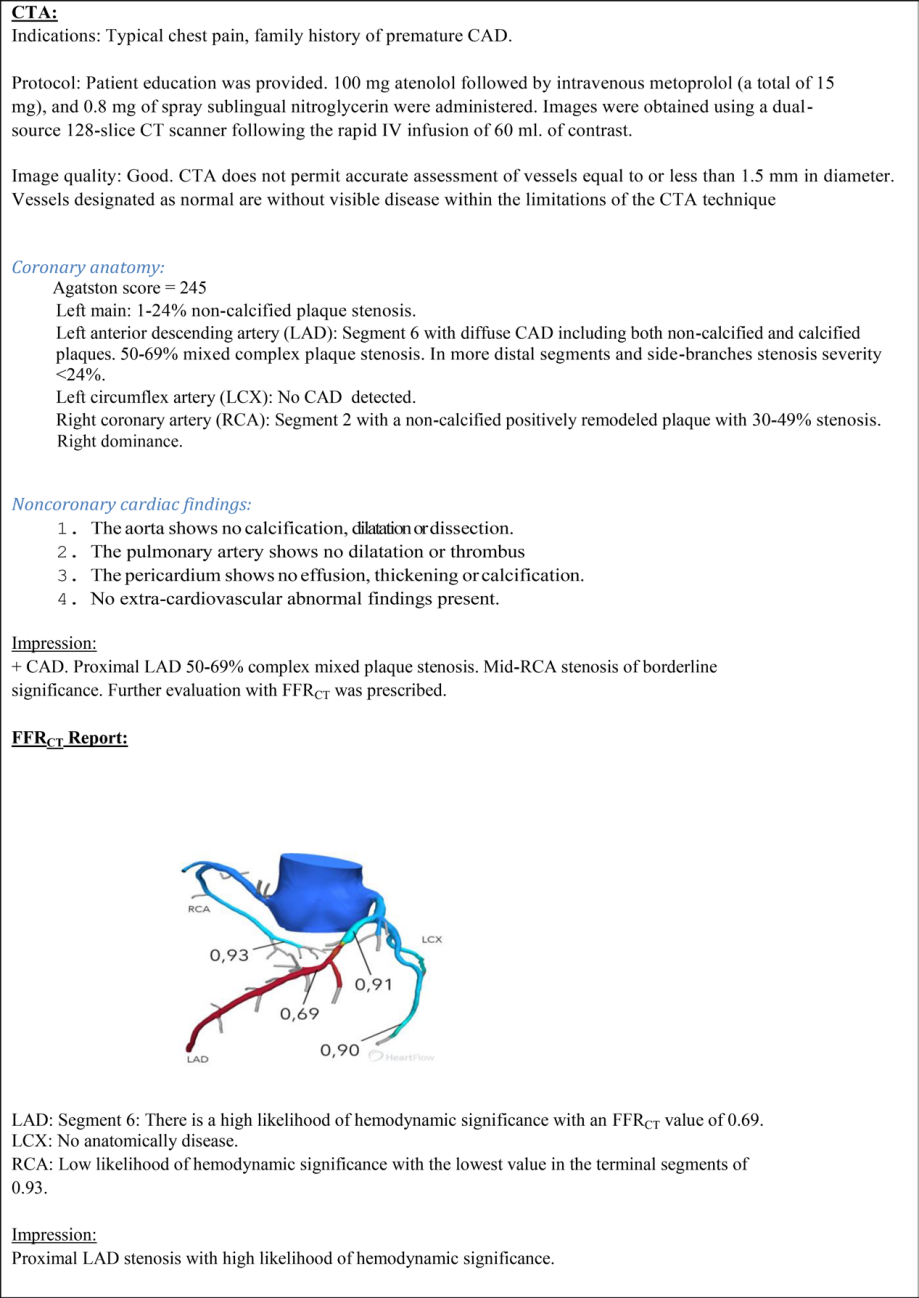
Example of a coronary CT angiography–FFR_CT_ report (patient case, Fig 1b). CAD = coronary artery disease, CTA = coronary CT angiography.

Coronary CT angiography and FFR_CT_ uniquely provide simultaneous anatomic and functional information in a noninvasive fashion. To provide useful, actionable guidance for medical or invasive management, the FFR_CT_ report must relate the observed anatomic coronary CT angiography findings with lesion-specific FFR_CT_ values. The principal purpose of the report is to communicate these findings and their clinical implications (Fig 4).

### Indications

The indications for the FFR_CT_ analysis should include clinical information from the original coronary CT angiography report, as well as specific anatomic details from the impression of the report that motivated the performance of FFR_CT_ analysis. Mention should be made of factors pertinent to the FFR_CT_ indication and suitability for analysis, such as angiographic degree of stenosis, extent of calcifications, and overall image quality (signal-to-noise ratio, motion artifacts, luminal contrast opacification). The indications should specify the anatomic lesions from the original coronary CT angiography report that were of particular concern in ordering the FFR_CT_ analysis. The present author group finds FFR_CT_ testing appropriate in patients with intermediate anatomic stenosis. FFR_CT_ values may be less than or equal to 0.80 in lesions of less than 50% diameter stenosis. Physiologic characterization with FFR_CT_ may be relevant in a small proportion of such lesions when located in proximal coronary segments supplying a large myocardial territory because they may have prognostic implications (40). On the other hand, even high-grade anatomic lesions with stenosis severity greater than 70% or even greater than 90%, which are generally considered flow limiting, may overestimate the physiologic significance (41,42). Therefore, we commonly use FFR_CT_ testing in the setting of more severe anatomic disease and multivessel disease to help guide decision making on downstream catheterization and potential revascularization planning (Fig 2). As with any test, the appropriateness is often determined on a case-by-case basis and commonly related to many factors beyond stenosis severity (Fig 2). Finally, because the impact of coronary occlusion on the diagnostic performance of FFR_CT_ is unknown, we do not recommend FFR_CT_ analysis to be prescribed in such circumstances.

### Results

We recommend FFR_CT_ values to be reported for each major coronary branch by specific coronary segments (diameter greater than 1.8 mm) using the standardized SCCT guidelines for coronary segmentation classification (21), and that the values be related to specific lesions within a given segment.

Any lesion identified in the original coronary CT angiography report as a potential source of pressure loss should be specifically reported in the FFR_CT_ report and its standard SCCT coronary segment identified. A given FFR_CT_ value may have different therapeutic implications if located in a proximal segment as opposed to either a distal location or within a minor side branch (25,29,30). If no FFR_CT_ value of 0.80 or less was reported in a given artery territory, we recommend the lowest value for that territory be reported. It is not necessary to provide FFR_CT_ values greater than 0.80 for minimal (1%–24% stenosis) or mild (25%–49%) lesions unless located in the left main or proximal LAD or when containing high-risk plaque features, in which case FFR_CT_ values should be provided. Any lesion with an abnormal FFR_CT_ value should be reported even if not considered as a likely source of significant pressure loss in the original coronary CT angiography report. We recommend that an FFR_CT_ value be provided for all moderate (50%–69%) and all severe (>70% to 99%) stenoses.

FFR_CT_ values 0.80 or lower that are measured more than 2 cm beyond a lesion not causing a significant focal pressure loss (FFR_CT_ > 0.80) should be reported when present in large vessels. The clinical significance of FFR_CT_ values 0.80 or lower in the distal coronary tree remote from any focal lesion is unknown. These may be reported; however, it should be stated that the values are remote from angiographic stenosis and are of uncertain clinical significance.

In the event of serial lesions, we recommend that the value of FFR_CT_ 10–20 mm distal to each lesion should be reported. If this is not possible, FFR_CT_ values between lesions should be reported, including information on the distance between stenosis and the FFR_CT_ value.

Occlusion of small vessels that were overlooked in the primary CT angiography assessment (typically involving distal segments or small side branches) may be revealed by the FFR_CT_ analysis process. While this may or may not be clinically relevant, an occluded branch may have some slight impact on FFR_CT_ in the parent vessel. The impact will depend on the size of the branch relative to other vessels. Occluded segments should be identified and referenced.

In recognition of the fact that FFR_CT_ is a mathematically derived analysis rather than an actual measurement of flow and pressure, it is recommended that results be described as demonstrating low, borderline, or high likelihood of hemodynamic significance rather than ischemia (Table).

### Impression

The report summary should focus on the presence of a low, borderline, or high likelihood of hemodynamic significance of the lesions identified in the impression section of the original coronary CT angiography report. In addition, any other lesion that has a borderline or high likelihood of hemodynamic significance should be reported even if it was not identified in the original coronary CT angiography interpretation. In particular, areas of diffuse coronary disease that produce low FFR_CT_ values distal to the affected segments should be described.

### Format

It is recognized that institutional requirements may dictate the specific reporting format required. Ideally, the coronary CT angiography and FFR_CT_ reports can be combined into a single uniform report that will most clearly relate anatomic and functional information. However, it is important to interrogate the anatomy to assess the extent and severity of CAD to determine the need for FFR_CT_ analysis. Given the time gap between the coronary CT angiography and FFR_CT_ results, either a preliminary CT angiography report may be finalized after the FFR_CT_ results are available or an FFR_CT_ report may subsequently be added to the original coronary CT angiography report. Both of these formats closely incorporate the most detailed description of anatomy and functional significance with minimal repetition. If institutions require a separate free-standing report, additional details in the indications should be provided to emphasize the severity, morphology, and location of lesions suspected of causing flow limitation.

### FFR_CT_ Images

It is recommended that relevant images from the FFR_CT_ report should be included if technically possible to more accurately convey the location of FFR_CT_ values at a specific anatomic location. This will help other physicians understand the location and extent of the pressure loss and the location for potential confirmatory invasive FFR measurement and will facilitate medical or invasive treatment planning. Providing images combining FFR_CT_ values and their specific location can rapidly and succinctly convey the extent of pressure loss and facilitate therapeutic decision making more easily than if textual description was offered alone.

### Management Recommendations

The decision of whether FFR_CT_ interpretation reports should contain management recommendations (ie, consideration for invasive coronary angiography or optimal medical therapy alone) will be determined by local institutional practices. If management recommendations are typically included in reports, note should be made that FFR_CT_ values should not be considered in isolation but are integrated with clinical and other imaging factors such as symptoms, plaque morphology, and lesion location. This is particularly important in cases of borderline FFR_CT_ values between 0.76 and 0.80 (Fig 2).

## Limitations

The diagnostic performance and utility of FFR_CT_ has been studied only in patients suspected of having stable CAD. At present, the use of FFR_CT_ in patients with stents or bypass grafts, microvascular dysfunction, prior myocardial infarction, or suspected or known acute coronary syndromes cannot be recommended. FFR_CT_ analysis cannot be performed in all patients. Coronary CT angiography–related artifacts, such as motion, misalignment, low contrast, or blooming from coronary calcification, may impair the diagnostic reliability of CT angiography and FFR_CT_ (43–45). It is our experience that FFR_CT_ has high diagnostic performance in patients with coronary calcification. However, our experience with FFR_CT_ testing in patients with severe calcification (Agatston score > 1000) is limited, and in two previous studies demonstrating high diagnostic performance of FFR_CT_ in vessels and patients with high calcium scores, the number of such patients were low (44,45). In previous multicenter studies of FFR_CT_ diagnostic performance, CT angiographic images were not of sufficient quality for FFR_CT_ analysis in 11%–13% of patients (15,16), whereas in more recent single-center studies that assessed the clinical utility of FFR_CT_, less than 4% of the patients did not meet the image quality requirements (10–12,14).

## Conclusion

By virtue of the complexity of the FFR_CT_ analysis providing information on pressure and flow across the entire coronary tree, standardized criteria on interpretation and reporting of the FFR_CT_ analysis results are of crucial importance both in context of clinical adoption of the test and in future research. For assessment of the hemodynamic significance of lesions, we recommend using the FFR_CT_ value 10–20 mm distal to the lower border of the stenosis. For clinical decision making, we recommend a dichotomous interpretation strategy be considered only in lesions with FFR_CT_ greater than 0.80 or less than or equal to 0.75, whereas in patients with FFR_CT_ ranging between 0.76 and 0.80, additional risk stratification information is needed. The results of FFR_CT_ must always be evaluated in their clinical context, taking into account patient symptoms, the coronary anatomy, and suitability of revascularization.
